# Spatial and temporal risk mapping of human and porcine *Taenia solium* infections in Malawi: a systematic review and geostatistical approach

**DOI:** 10.1186/s42522-026-00199-3

**Published:** 2026-03-10

**Authors:** Nicholas Ngwili, Upile Kachepa, Salaviriuse Ahimbisibwe, Max Korir, Mercy Chavula, Catherine Wood, John Chiphwanya, Holystone Kafanikhale, Camille Glazer, Lazarus Juziwelo, Pemphero Munkhondia-Phiri, Janelisa Musaya, Lian Francesca Thomas, Matthew A. Dixon

**Affiliations:** 1 Health Program, International Livestock Research Institute, Nairobi, Kenya; 2 Department of Animal Health and Livestock Development, Lilongwe, Malawi; 3 Lilongwe University of Agriculture and Natural Resources, Lilongwe, Malawi; 4 Ministry of Health, Lilongwe, Malawi; 5 University of Liverpool, Liverpool, United Kingdom; 6 Queen Elizabeth Central Hospital, Blantyre, Malawi; 7 Kamuzu University of Health Sciences, Blantyre, Malawi; 8 Royal (Dick) School of Veterinary Studies, University of Edinburgh, Easter Bush Campus, Edinburgh, EH25 9RG United Kingdom; 9 Department of Infectious Disease Epidemiology and London Centre for Neglected Tropical Disease Research (LCNTDR), Faculty of Medicine, School of Public Health, Imperial College London, London, UK; 10 Unlimit Health, Edinburgh House, London, UK; 11 College of Veterinary Medicine, Animal Resources and Biosecurity, Makerere University, Kampala, Uganda

**Keywords:** Taenia solium, Risk factor mapping, Spatial statistics, One health, Zoonotic diseases

## Abstract

**Background:**

*Taenia solium,* colloquially called the pork tapeworm, is a zoonotic parasite with a human definitive host and a porcine intermediate host. Humans can become an aberrant intermediate host due to accidental ingestion of parasite eggs from the environment or through autoinfection, resulting in human cysticercosis (HCC), or neurocysticercosis (NCC) if the central nervous system is infected. Pigs become infected with the larval stage, porcine cysticercosis (PCC), through the ingestion of parasite eggs shed by humans through defecation. Malawi has been classified as endemic for *T. solium* by the WHO based on the presence of key risk factors; however, the subnational distribution is not known. To ensure the appropriate resources are mobilized to support targeted future *T. solium* control measures in Malawi, there is a need to understand the variation in *T. solium* endemicity status across the country.

**Methods:**

The current study uses a systematic literature review (SLR) using a pre-registered protocol; (PROSPERO CRD42023411044) to collate all available evidence on *T. solium* in Malawi. A geospatial risk mapping approach was conducted based on data from Malawi Demographic and Health Surveys (MDHS), and pig density data from the Food and Agriculture Organization (FAO) database to create geospatial risk maps of endemic subnational areas for 2000, 2004, 2010, and 2016. To create a single composite risk factor map for the four years from the MDHS, each parameter was plotted as a binary variable with the high or low risk categories and overlaid into a single composite risk factor classification. Additional data from hospital records on NCC and meat inspection records across several Agricultural Development Divisions (ADDs) were also collected.

**Results:**

A total of 9 studies were identified through the SLR, with 8 studies focused on HCC, including one study studying both HCC and PCC, and one study on PCC. The review of the hospital reports captured 38 reports of NCC, with the cases being from around the location of the two major hospitals. PCC rates in slaughter animals substantially varied among the six ADDs, with the highest rates being in Mzuzu ADD, with 3.7 cases per 100 ps-years^-1^, and in Salima ADD, with 4.5 cases 100 ps-years^-1^. Geospatial risk mapping results for the years 2000 to 2016 indicated that PCC risk factors exhibited substantial geographic and temporal variation. Areas at highest risk, with the presence of all three risk factors, were predominantly found in the central and southern districts of Malawi across years. High pig density and poverty in combination were evident across Malawi in 2000; however, these areas declined in northern districts between 2004 and 2016 while remaining consistent in central districts. The combined map from the four data streams did not provide validation for the risk maps but provided more robust evidence about the potential endemicity of the parasite in Malawi.

**Conclusions:**

Most studies identified from the SLR were case reports and mostly focused on HCC, with very limited literature available in terms of population-based studies. The confluence of high levels of poverty, poor sanitation, and high pig densities is evident in central and southern districts, indicating potential *T. solium* endemic areas warranting further investigation. Review of NCC cases from hospital records and PCC rates in slaughtered animals across ADDs further supports prioritizing these areas. Targeted epidemiological and socio-economic studies to inform interventions in the possibly hyperendemic areas are urgently required.

**Supplementary information:**

The online version contains supplementary material available at 10.1186/s42522-026-00199-3.

## Introduction

*Taenia solium* is a zoonotic cestode, with humans as the definitive host and pigs as the intermediate host. Humans can become an aberrant intermediate host (human cysticercosis; HCC) following accidental ingestion of parasite eggs from the environment or through autoinfection. Upon consumption, the eggs develop into cysticerci (larval-stage) and migrate to the central nervous system, which can result in human neurocysticercosis (NCC) [1], [2]. The main transmission cycle propagates after humans consume undercooked pork infected with cysticerci [3]. In the intestine, *T. solium* adult tapeworms develop, resulting in human taeniasis (HT), giving the parasite its colloquial name, the ‘pork tapeworm’. Pigs become infected with the larval stage (porcine cysticercosis; PCC) through the ingestion of parasite eggs or proglottids containing eggs, which are shed by humans following defecation, particularly in areas where latrine use is uncommon or latrines are poorly constructed [4–6]. The cysts lodge in the muscle and subcutaneous fat layer of the pig, causing PCC [4].

NCC-mediated epilepsy, which renders people incapacitated, unproductive, and sometimes leads to fatal accidents because of seizures, is the most common preventable cause of seizures in the developing world [7, 8]. Globally, it is estimated that NCC results in approximately 50,000 deaths per year [9]. Over 80% of the people affected by epilepsy live in low- and middle-income countries (LMICs), with 1.90 to 6.16 million cases originating from sub-Saharan Africa [10–12]. At a global level, *T. solium,* which remains neglected, accounts for over 1.18 million disability-adjusted life-years (DALYs) in 2023 [13]. In addition, PCC affects the health, welfare, and productivity of the pigs, further hampering efforts to eradicate poverty and hunger [8, 14, 15]. The WHO NTD 2021 – 2030 Roadmap calls for the control of *T. solium* to be intensified in hyperendemic areas of the endemic countries (WHO, 2020). Countries are not currently on target to meet the 2030 milestones, given the lack of national control programmes to tackle *T. solium*, and the lack of definition for what constitutes “intensified control”.

The spatial distribution of *T. solium* has been characterized for Tanzania [16] and Uganda [17], while its co-distribution with schistosomiasis has also been described for the Sub-Saharan Africa (SSA) region [18]. More work is yet required to assess the distribution of infection indicators and risk factors in other SSA countries, particularly to identify hyperendemic subnational areas. In Malawi, pig farming has emerged as a promising agricultural sector, contributing to the country’s food security, economic growth, and poverty alleviation, with the pig population growing from 2,433,172 to 7,355,254 between 2012 and 2019 [19]. Rising demand for pork in Malawi will, however, present biosecurity challenges to both animal and human health [19], due to increasing pig densities and risk for disease outbreaks. Malawi has been classified as endemic for *T. solium* by the WHO, based on the number of pigs, reduced access to sanitation, type of pig production, proximity to other endemic countries and predominant religion [20]. Unlike African Swine Fever (ASF), which has been extensively reported in Malawi since 1989 [21], little published data on *T. solium* exists at the sub-national level for Malawi [22, 23]. To ensure resources are mobilized and directed to where *T. solium* surveys are needed and ultimately target control measures in Malawi, there is a need to understand the variation in *T. solium* risk and endemicity across the country. The current study provides a novel contribution to the literature by combining the use of a systematic literature review (SLR) to collate all available evidence on *T. solium* in Malawi, including a review of hospital records to obtain information on NCC and unpublished regional meat inspector data on PCC cases, and complemented by a geospatial mapping analysis of risk factor distribution across the country.

## Materials and methods

The study uses two approaches, namely an SLR (pre-registered protocol: PROSPERO CRD42023411044) to collate all published and unpublished data on *T. solium* infections and geospatial risk mapping using indicators extracted from the Malawi Demographic and Health Surveys (MDHS) and pig density data from the Food and Agriculture Organization (FAO) database. The SLR was conducted following the Preferred Reporting Items for Systematic Literature Reviews and Meta-analysis (PRISMA) guidelines [24]. The PRISMA checklist has been provided as an additional file, Table 2. PRIMSA checklist.

The PICOS (participants, interventions, outcomes, study designs) framework was used to structure the SLR. The time horizon included any relevant studies conducted in Malawi up to 9^th^ September 2022, when the search was done with no lower limit.

The protocol and the full data extraction tool with full details on the search strategy, including the PICOS framework, eligibility criteria, inclusion criteria, and search terms used in PubMed, African Journals Online (AJOL), Cabdirect, OVID Medline, Web of Science, Cochrane Library, and Google Scholar are available in the additional file Text S1.

### Calculation of informed prevalence estimates and uncertainty

All analyses were conducted using R for statistical computing version 4.2.2. Where information on the number of positive and total sampled was available from studies, binomial confidence intervals were calculated with the R ‘prevalence’ package [25]. Observed prevalence from the literature were further recalculated to estimate informed prevalence with a Bayesian framework by incorporating sensitivity and specificity estimates for the respective diagnostics (using the ‘prevalence’ package in R [25]). The 95% Bayesian credible intervals were calculated from the posterior distributions of informed prevalence. Uniform distribution priors for diagnostic specificity and sensitivity were specified (see additional file, Table 1 and associated text for further details). Available informed prevalence estimates were then mapped in R.

### Collection of additional unpublished data from Malawi

**Porcine cysticercosis data**: records made by meat inspectors on the numbers of pigs inspected and their disease status were collected from all Agricultural Development Divisions (ADDs). Statistical analyses were carried out to obtain PCC prevalence estimates adjusted for diagnostic sensitivity and specificity, as highlighted in the ‘Calculation of informed prevalence estimates and uncertainty’ section. Data were collected via formal and informal requests from the government and private sectors involved in pig slaughter. The Department of Animal Health and Livestock Development did not have a central database of requested data, but gave permission to obtain all available data from the regional heads of the 6 ADDs in Malawi. Available data included the number of pigs slaughtered and the number of carcasses condemned due to the presence of cysts. Lilongwe, Machinga, Mzuzu, Kasungu, Blantyre, and Salima ADDs were able to provide data for the ADD, while records at the sub-ADD level in the Extension Planning Area (EPA) were also available for Mzuzu and Salima (years 2020–2023). Records for the other ADDs were available for one year between July 2020 and June 2021. To allow comparison among datasets provided from ADDs, all available data were converted to a standardized measure of cysts present per 100 pigs slaughtered per year (per 100 ps-years^−1^).

**Neurocysticercosis data from hospital records**: A review of hospital records was conducted in the two main referral hospitals, Queen Elizabeth Central Hospital (QECH) in Blantyre and Kamuzu Central Hospital (KCH) in Lilongwe. The aim was to gather more data on HCC/NCC as captured through hospital reports of patients diagnosed either clinically or through laboratory diagnosis. Although the International Classification of Diseases (ICD)–10 are not standardized for Malawi, records were retrieved from the radiology department and the information on age, sex, district of origin, diagnosis and method or diagnosis collected. The data was collected through Open Data Kit (ODK) and uploaded to ILRI servers for further summarizing.

### Risk factor mapping

Data processing and analysis were performed using R version 4.2.2 to generate PCC risk maps for 2000, 2004, 2010, and 2016, using the MDHS. The proportion of two PCC risk factors for each MDHS cluster was estimated, including the proportion of households in the lowest two socio-economic quintiles (poverty variable) and the proportion of households with no toilet facility, or specified as using bush or field (sanitation variable), shown in Table 1. Semi-variograms for each risk factor were computed to assess the degree of spatial autocorrelation, with spherical or wave variogram models fitted to these data (additional file Figure 5 presents model fits). The ordinary kriging technique was then applied to interpolate and predict the proportion of households with the specific variables across Malawi, generating a smooth map of predicted risk factor values at unsampled locations. Based on each variable’s upper third (66th percentile) distribution of the data (Table 1), the interpolated data points were classified as high or low risk. For the third risk factor, a smooth pig population density map at 1 km spatial resolution in Malawi was used [26]. Each risk factor was weighted equally due to the absence of robust estimates of its relative contribution to *T. solium* transmission. To create a single composite risk factor map for the four MDHS factors in the composite maps are grouped into three levels based on poor sanitation (Risk Factor A), high pig density (Risk Factor B), and high levels of poverty (Risk Factor C), as well as combinations of these factors. An additional map was produced by combining the data from the SLR, PCC data from the ADDs, NCC data from the review of hospital records and the risk mapping data for 2016 to provide a holistic picture of what is currently known about the epidemiology of the parasite in Malawi.

**Table 1:** Data sources for risk factor mapping

Year	Risk factors & data sources		Threshold values define the proportion of the population between low/high	
	**Sanitation**^**a**^ **& poverty index**^**b**^	**Pig population density**^**c**^	Poor Sanitation	Poverty
2000	Malawi Demographic and Health Surveys (DHS) (USAID, 2000)	Modelled livestock densities from the Gridded Livestock of the World Database 2007 (Wint & Robinson, 2007)	0.384	0.338
2004	DHS (The DHS Program, 2004)		0.441	0.728
2010	DHS (USAID, 2010)		0.408	0.666
2016	DHS (USAID, 2016)		0.885	0.675

^a^household with no toilet facility/or specified as using a bush or a field

^b^household-level poverty indicator classified as those households in the lowest two socio-economic quintiles

^c^Pig densities with threshold set to ≥ 1 pig per km^2^,as included in Robinson *et al*. [26]

## Results

### Systematic literature review results

**Fig. 1:**
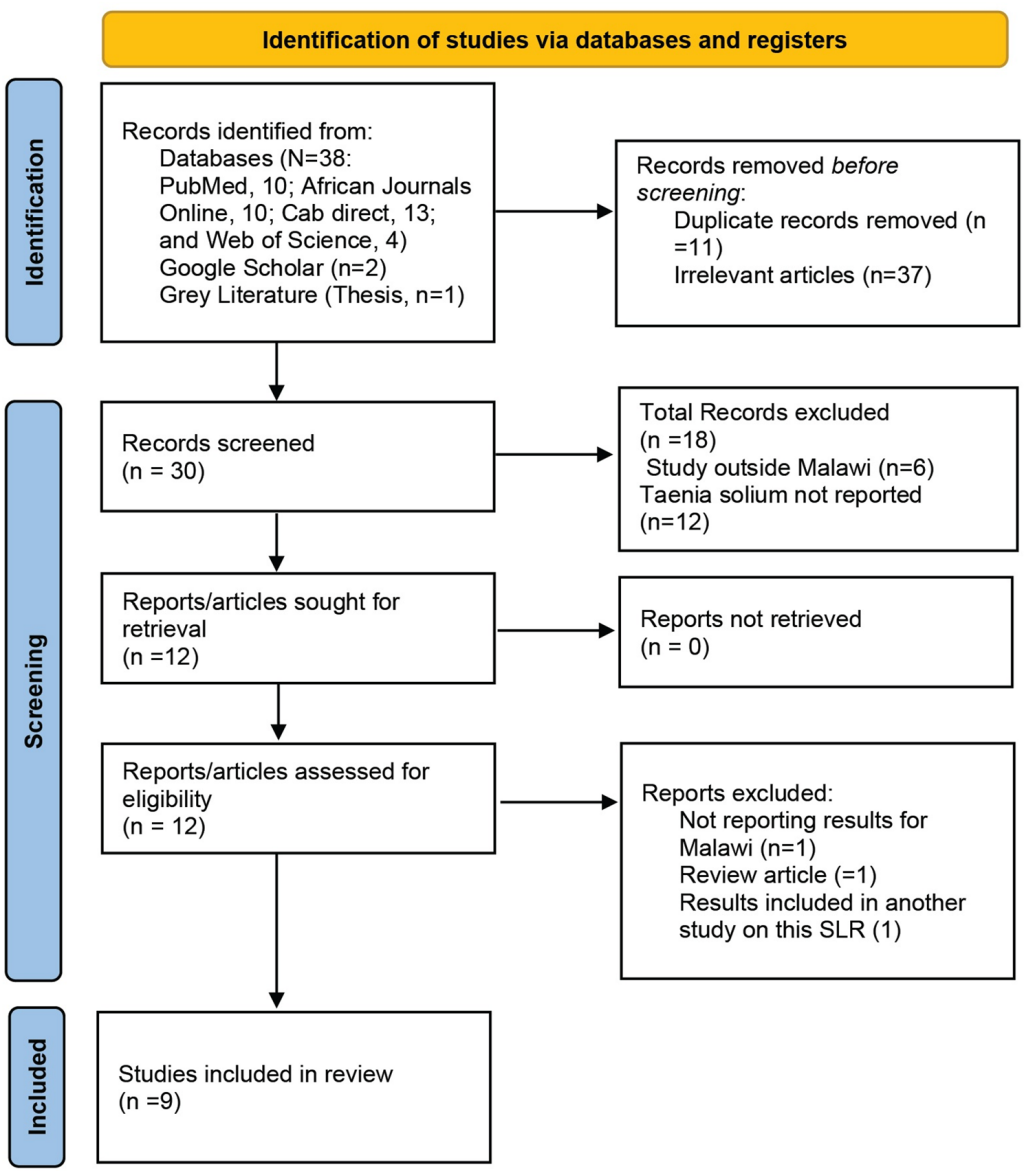
PRISMA flow chart applied to the systematic literature review for identifying *T. solium* studies

A total of 9 studies were identified through the SLR as shown in the PRISMA flow diagram (Fig. 1). Eight studies measured HCC, and one study measured PCC. A total of 3 studies were population-based and 6 were case reports, with all studies conducted between 1992 and 2022.

**Fig. 2:**
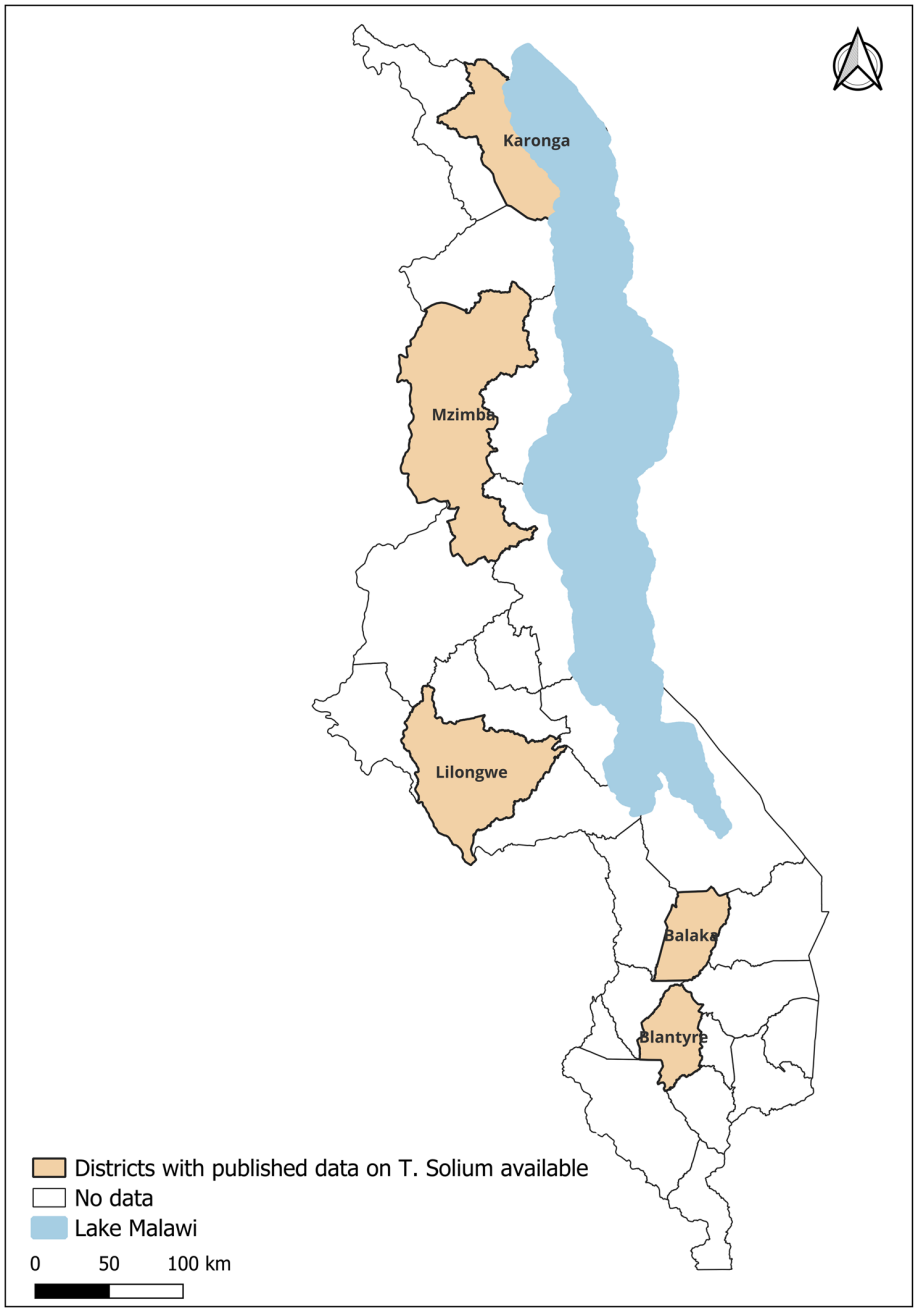
Distribution of published studies in Malawi by district

Across the case reports, NCC was more commonly reported than subcutaneous HCC, with imaging techniques encompassing CT scans, MRI, and ultrasonography utilized in 5 studies and serology using the Enzyme-linked Immunoelectrotransfer Blot (EITB) in one study (Table 2). Two case reports identified HCC in patients located outside of Malawi but having previously resided in Malawi [27, 28] (Fig. 2). In both instances, the patients were initially diagnosed with epilepsy and schizophrenia before subsequently being diagnosed with NCC. Five reported favorable outcomes, with patients surviving their respective conditions. In one of these studies, Bills and Simon [27], the patient experienced long-term side effects. In the one study in which the patient died [29], the patient was originally diagnosed with HIV-associated cryptococcal meningitis and only officially diagnosed with NCC postmortem. The limitations of these studies have been provided in the additional file, Table 2.

**Table 2:** Case report studies included in the SLR

Reference	Year	Recruitment	Location of the study	Manifestation of the disease	Diagnostic method used	Outcome/ Prevalence
Bills et al.	1992	Patient presenting to hospital	London, UK*	Neurocysticercosis	CT scan and immunofluorescence	Alive
Ponnighaus et al.	2001	Patient presenting to hospital	Karonga	Subcutaneous cysticercosis	Histopathology	Alive
Uledi SJ	2010	Patient presenting to hospital	Mzuzu	Subcutaneous cysticercosis	X-ray, MRI, Ultrasonography Histopathology	Alive
Dhesi et al.	2015	Patient presenting to hospital	Coventry, UK*	Neurocysticercosis	MRI	Alive
Heller et al.	2017	Patient presenting to hospital	Lilongwe	Neurocysticercosis & Subcutaneous cysticercosis	Ultrasonography, CT scan	Alive
Kalata et al.	2021	Patient presenting to hospital	Blantyre	Neurocysticercosis	MR, Lumbar puncture, EITB	Died

An asterisk (*) next to the location indicates the patient emigrated from Malawi

The population-based studies (*n* = 3) included two cross-sectional surveys and one cohort study (Table 3). One study reported NCC prevalence of 4.4% (95% Bayesian credible interval (BCI), 0.8–8.5%) based on antigen and antibody ELISA and a CT scan [30], and one study reported PCC prevalence of 3.5% by tongue palpation (TP) and 5.3% by routine meat inspection (MI), informed prevalence of 17.9% (95% BCI 0.9–67.4%) for TP, and 14.0% (95% BCI 0.7–47.0%) for MI [31]. The other study investigated the differential diagnosis of stroke in patients presenting with stroke and found that 1 out of 98 patients had NCC [32].

**Table 3:** Population-based studies included in the systematic literature review

Reference	Year	Study type	recruitment	Location of the study	Manifestation of disease	Diagnostic method	Outcome/prevalence
Kumwenda et al.	2005	Cohort study	Patient recruitment	Blantyre	NCC	CT scan	lost to follow up
Keller et al.	2022	Cross-sectional	Door-to-door recruitment	Balaka	NCC	EITB, Ag-ELISA, CT scan & MRI	*4.4% (0.8–8.5%) in PWEs
Banda L.	2019	Cross-sectional	Random sampling	Blantyre & Lilongwe	PCC	Palpation and incision methods	3.5%;**17.9% (0.9–67.4%) TP 5.3%, **14.0% (0.7–47.0%) MI

TP = Tongue palpation, MI = Meat inspection and PWE = Person with epilepsy, *Adjusted prevalence calculated in the published study (Bayesian credible interval (BCI) calculated by study), ** Informed prevalence calculated in this analysis (95% BCIs)

### Additional primary data on porcine cysticercosis

**Fig. 3:**
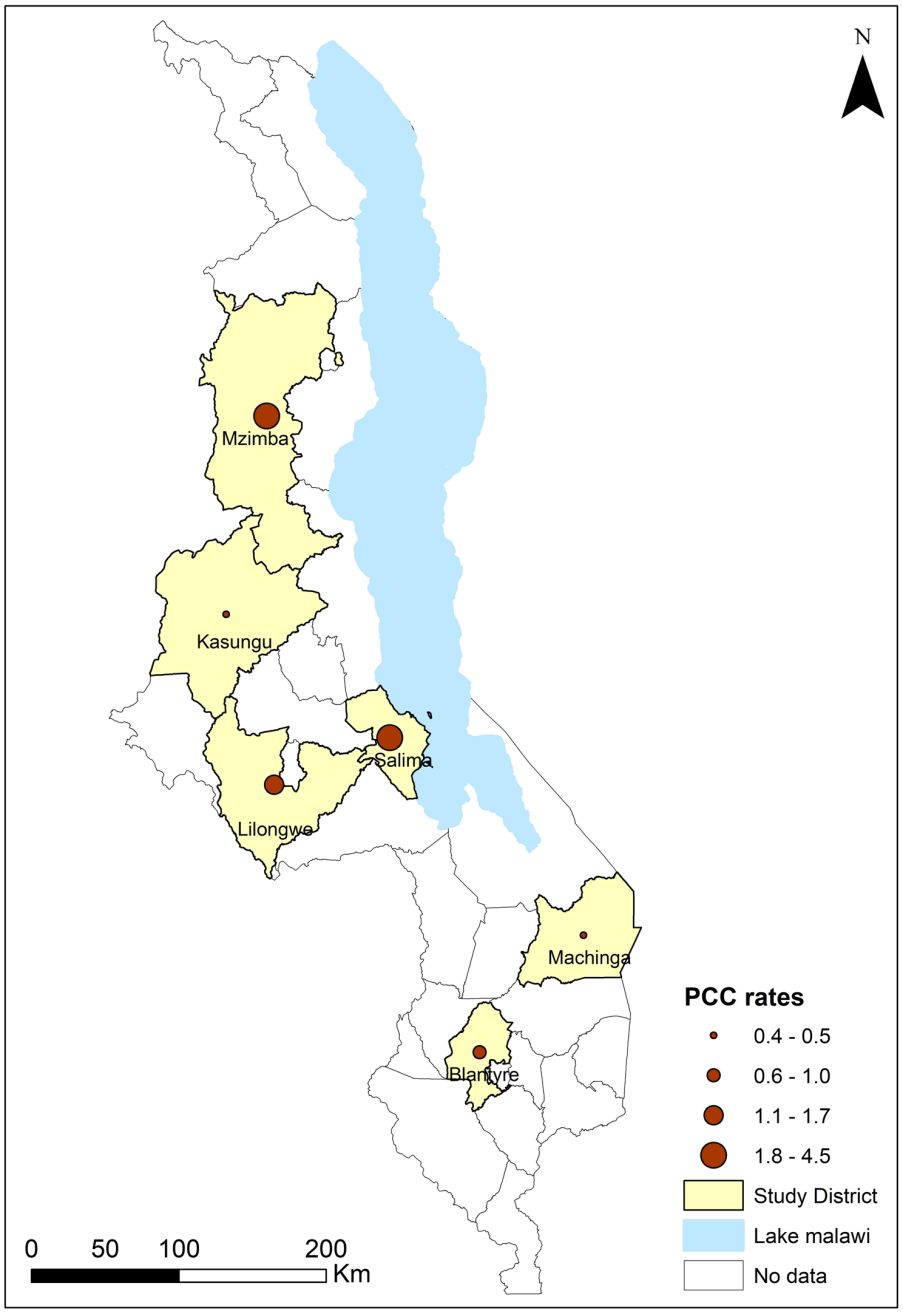
PCC rates in slaughtered pigs from six Agricultural Development Divisions. Rates are expressed as cases per 100 pigs slaughtered per year for each ADD. For Salima and Mzuzu (found in Mzimba district), rates were available for three years, so the range is presented. In addition, the HCC and PCC prevalence values have been plotted based on the location of the respective study. The maps were generated using QGIS 3.28.5.

PCC rates in slaughter animals substantially varied among the six ADDs drawn from different districts (Fig. 3). The highest rates were found in Mzuzu ADD (found within Mzimba district) with 3.7 cases per 100 ps-years^−1^ and in Salima ADD with 4.5 cases 100 ps-years^−1^ (Fig. 3). Salima ADD consists of two administrative districts; Salima District had a higher rate of 4.5 cases per 100 ps-years^−1^ whereas Nkhotakota District had a lower rate of 3.7 cases per 100 ps-years^−1^. The primarily urban ADDs of Lilongwe and Blantyre had rates of 1.7 cases per 100 ps-years^−1^ and 1.0 cases per 100 ps-years^−1^, respectively. Kasungu and Machinga ADDs had the lowest rates with 0.5 cases per 100 ps-years^−1^ and 0.4 cases per 100 ps-years^−1^, respectively. It should be noted that despite the large pig populations, relatively few slaughters were recorded in Machinga, while larger numbers of slaughters were reported in Lilongwe and Blantyre.

Sub-ADD rates within Extension Planning Areas (EPAs) were available for Salima and Mzuzu, remaining consistent over 2020–2023. Some EPAs had consistently higher rates, with 16.9 cases per 100 ps-years^−1^ − 30.5 cases per 100 ps-years^−1^ in Katowo EPA (Mzuzu ADD) and 4.3 cases per 100 ps-years^−1^ − 11.9 cases per 100 ps-years^−1^ in Mtosa EPA (Salima ADD). Matenje and Katelera EPAs (Salima ADD) also had rates of 10.0 cases per 100 ps-years^−1^ or higher for years with available data (2021 and 2022), although the sample sizes were small.

### Additional primary data on neurocysticercosis from hospital records

**Fig. 4:**
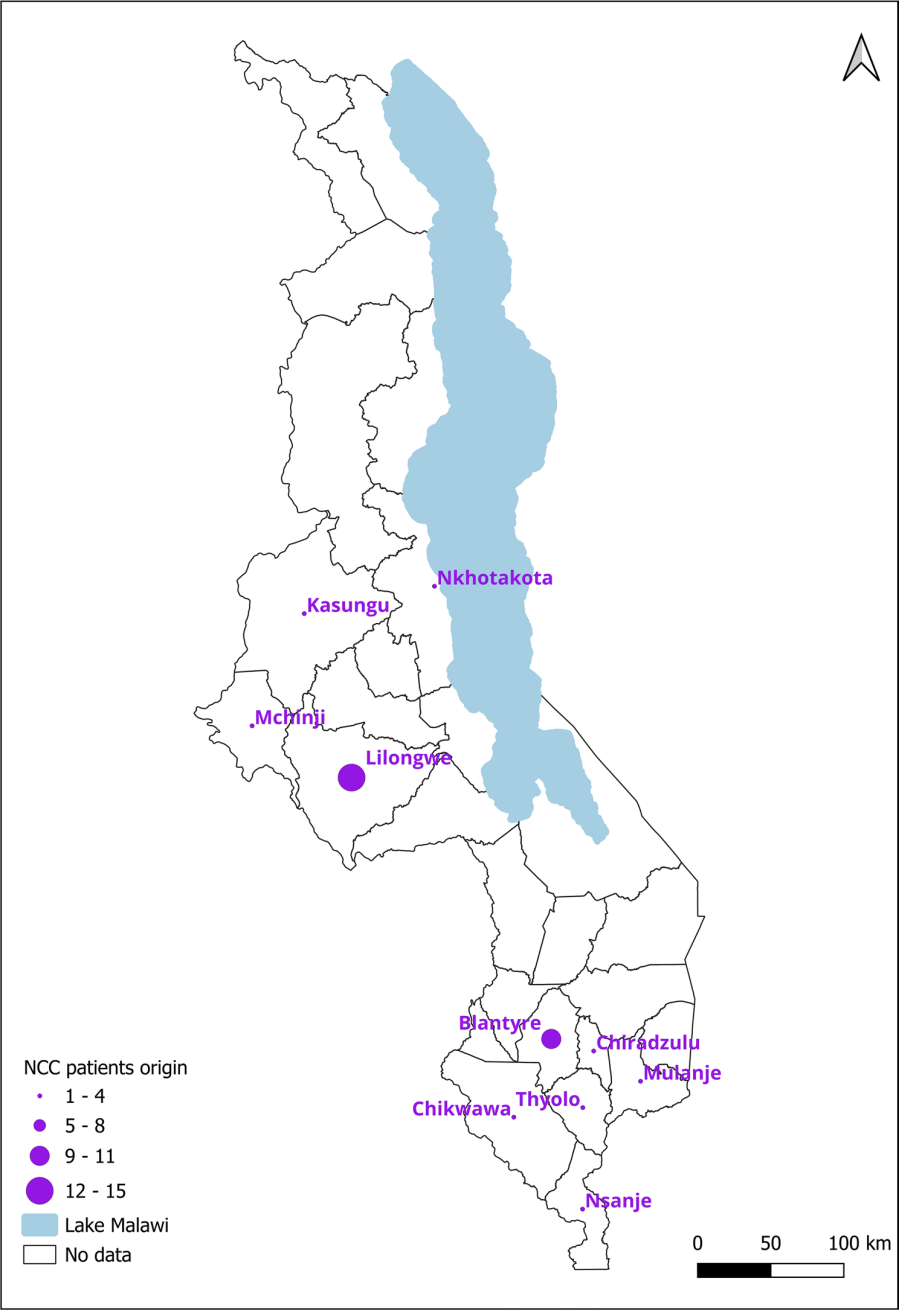
Districts of origin for NCC patients diagnosed at QECH in Blantyre and KCH in Lilongwe

A total of 38 hospital records recorded between 2013 and 2024 were reviewed from both QECH in Blantyre and KCH in Lilongwe. In both hospitals, older records were not available because of the arrangements of the paper-based filing system. The majority of the patients were male (*n* = 28, 73.68%) as compared to female (*n* = 10, 26.32%). The mean age was 39.5 (standard deviation of 17.8), with a minimum of 16 and a maximum of 86. Most NCC cases were diagnosed based on brain CT scans, with some diagnoses also made using lumbar puncture, particularly in QECH. Out of the 38 patients, 24 of them presented with convulsions and headaches, while 13 had seizures. Other symptoms included confusion, dizziness, chills, tongue biting and eye rolling. The patients’ areas of origin were clustered around the two main hospitals. Most of the patients presenting at the two hospitals originated from districts not far from the hospital, with a few exceptions (*n* = 3) being those originating from the middle region of Malawi and *n* = 5 cases from the southern tip of Malawi (Fig. 4).

### Spatial risk mapping results

**Fig. 5:**
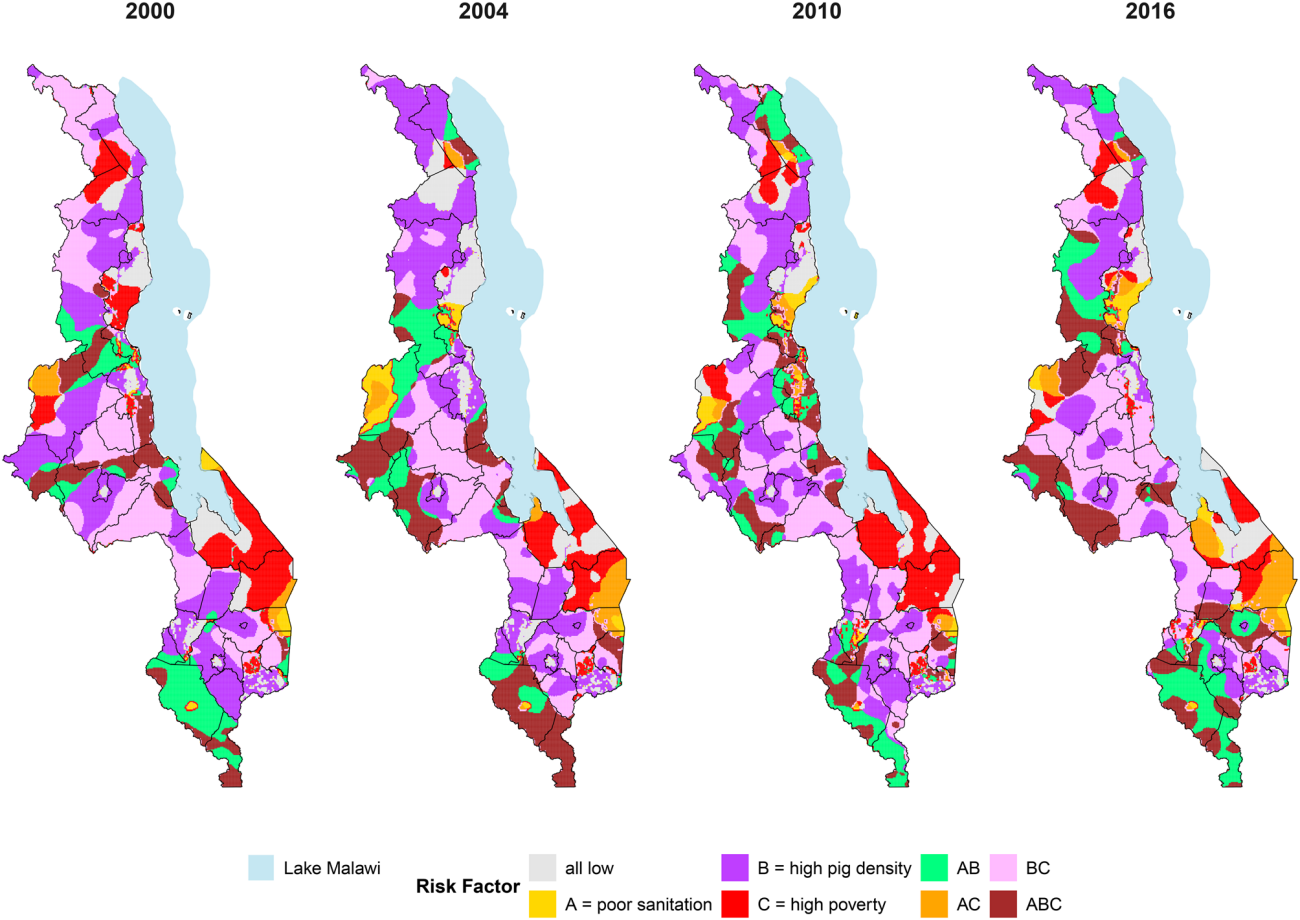
Distribution of porcine cysticercosis risk factors between 2000 and 2016 in Malawi. Risk factors include low sanitation (**A**), high pig density (**B**), and high poverty (**C**), with maps showing the distribution and confluence of these risk factors. Lake Malawi is also plotted. Districts presented are relevant for 2018

During the period spanning 2000 to 2016, PCC risk factors varied substantially geographically (Fig. 5). Areas at highest risk, with the presence of all three risk factors were predominantly found in the central and southern districts of Malawi (brown areas), with some pockets further north from 2004 to 2016. A combination of high pig density and poverty was evident across Malawi in 2000 (pink areas); however, these areas declined in northern districts between 2004 to 2016, while remaining in central districts. Pockets of combined poor sanitation with pig density were found throughout Malawi between 2000 to 2016 (green areas), becoming more prevalent in southern areas in 2016. Poor sanitation (yellow colour) appeared to be prevalent in areas on the shores of Lake Malawi in 2010 and 2016, with the addition of a high poverty pocket in the southeastern part of the country in 2016 (green area). In three eastern and central districts in 2016, an area of combined poor sanitation with poverty emerged (orange areas). However, the lack of high pig densities means this area was not considered a high priority for PCC risk. Additional files: Figs. 1–4 show the individual risk maps for each variable by year.

### Combined mapping using the four different data streams

**Fig. 6:**
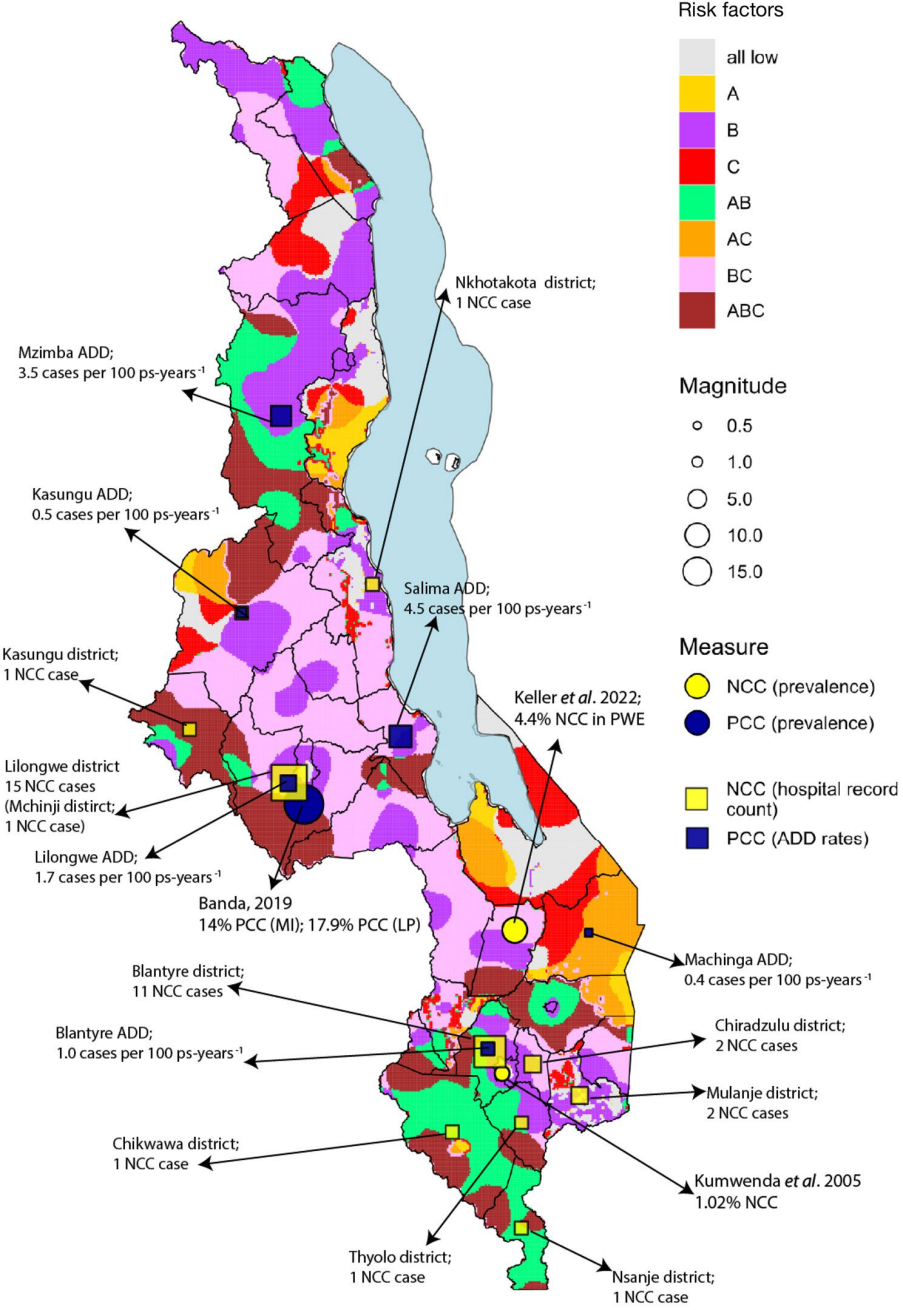
Combined map based on all 4 data streams (SLR data, PCC data from ADDs, NCC data from hospital records reviews and risk map for 2016)

A combined map was generated by combining the SLR data, PCC data from ADDs, hospital record data and the geospatial risk mapping for the most recent year (2016) (Fig. 6). This map indicates key high-risk zones (i.e. ABC) in the south, to the west of Lilongwe, towards the Chipata border crossing and the western border between Kasungu and Mzimba districts, although the latter two areas do not have published survey or ADD/hospital record-based data. The published data and record data originate predominantly from areas near the major urban settlements of Lilongwe and Blantyre, with only one published data source originating from a probable high-risk zone on the border areas of Mchinji district.

## Discussion

This study is the first to compile data from multiple sources and spatial mapping approaches to understand the epidemiological landscape of *T. solium* in Malawi. Unlike earlier reviews undertaken in East Africa, this analysis synthesizes diverse and complementary data sources to provide a more holistic understanding of the baseline epidemiology of *T. solium* in Malawi. Notably, it is the first to integrate hospital records of NCC, thereby expanding the evidence base beyond community and field surveys and demonstrating the value of incorporating additional data streams when information from the published literature is limited.

All but one study identified from the SLR contained information on HCC, with the majority being case reports. Only 3 population-based studies were identified, with only one study measuring PCC prevalence, highlighting a clear research gap. The availability of 2 NCC studies, however, is encouraging, as the condition remains substantially under-represented in literature. These studies considered NCC within 2 specific populations, being people with epilepsy [30] and stroke patients [32]. Neighboring countries such as Zambia have undertaken extensive research to estimate prevalence, identify risk factors, and implement control strategies related to *T. solium* [33]. The highly endemic eastern region of Zambia remains an area of concern for Malawi, due to the potential risk of cross-border transmission. Other country-specific mapping reviews have found a greater number of population-based studies. In Uganda [17], a total of 16 population-based studies were identified, while in Tanzania, a total of 57 population-based studies were captured by a scoping literature review, including 4 intervention studies [16].

These differences may stem from the variations in capacity within the country and the interests of long-term collaborators. *T. solium* research has long been driven by the veterinary community, and Malawi opened its first vet school in 2014, unlike Uganda, which established its first veterinary school in 1971 [34], Tanzania in 1976 [35] and Zambia in 1983 [36]. Long-term research collaborations between university of Zambia and Institute of Tropical Medicine (ITM) and Ghent University, Sokoine University of Agriculture and University of Copenhagen and ITM, Gulu University & Technical University of Munich (TUM), Makerere University & ILRI have all driven extensive research on *T. solium* in these countries. These long-standing collaborations provided a potential model for supporting Malawi in building research initiatives aimed at fully characterizing the baseline epidemiology of *T. solium* and evaluating the feasibility, acceptability, and effectiveness of locally appropriate control options. Moreover, the countries highlighted above have, over the years, strengthened their veterinary health capacity and associated systems, offering an important foundation upon which to advance such research efforts. Similar collaborative efforts have started between ILRI and LUANAR where veterinary students have been supported to undertake their 5^th^ year research projects through funding and supervision by ILRI for the last four years [37].

Our SLR identified case studies reporting both NCC and subcutaneous HCC in Malawi, with diagnosis of these conditions primarily relying on neuroimaging and ultrasonography techniques. Notably, two cases of NCC were diagnosed in the United Kingdom in patients who were originally from Malawi, highlighting the potential risk of imported infections associated with migration. This also potentially highlights the limited access to diagnostics and treatment in Malawi, with techniques such as neuroimaging restricted to major referral hospitals, which likely leads to a severe underestimation of HCC/NCC in Malawi. Missed or misinterpreted diagnoses can have detrimental effects on the patients’ health outcomes, resulting in delayed treatment, prolonged morbidity, and potential excess mortality.

Only one study reported PCC in the literature with data, collected at the point of slaughter using meat inspection [31]. The data reported in this study were obtained from the region around Mchinji district, which is likely to be a high-risk area, as indicated by the risk mapping. We therefore recommend further cross-sectional studies guided by the risk map and the gaps in the current literature to confirm the endemicity of the parasite across Malawi. The use of meat inspection in the single published PCC study may have led to an under-estimation of the PCC prevalence due to its low sensitivity, especially in detecting light and moderate intensity infections in pigs [38]. This necessitates adjusting observed prevalence to generate informed prevalence estimates, as undertaken in this study. However, the uncertainty in the diagnostic performance of both lingual palpation and meat inspection in the Banda [31] study—combined with the small sample size—resulted in wide Bayesian credible intervals around the estimated true prevalence. This reiterates the need for more studies using diagnostic techniques with better sensitivity and specificity. The accuracy of meat inspection relies heavily on the expertise of inspectors and the thoroughness of the examination process [38]. In Malawi, Banda et al. [31] further reports that some meat inspectors did not feel obliged to conduct proper meat inspection, despite regulations mandating inspection before meat enters the food chain. This practice could be related to insufficient training of meat inspectors and a lack of central slaughter places for pigs in the country. Additionally, a shortage of resources, such as time constraints and understaffing, could also hinder their ability to conduct thorough inspections. This highlights the need for retraining and retooling of meat inspectors to increase the efficiency of the meat inspection processes. This situation is not unique to Malawi, but common in most East and Southern Africa countries, for example, in Uganda [39], in Tanzania [40] and in Zambia [33].

Malawi has a comprehensive legislative and policy framework concerning livestock and public health, encompassing the Livestock Policy, the Meat and Meat Products Act [1975] [41], the Control and Diseases of Animals Act [1967] [42], the National Sanitation and Hygiene Strategy 2018, the National Sanitation Policy 2008 [43] and the Protection of Animals Act [Cap. 66:01] [44]. Collectively, these serve to emphasize the importance of farming practices, meat inspection, disease surveillance, and sanitation measures to reduce the spread of zoonotic diseases such as *T. solium*. However, despite the presence of these regulatory documents, the risk mapping component of our work highlights the presence of risk factors that continue to perpetuate transmission of *T. solium*. Notably, there has been an expansion of geographic areas with all three risk factors from 2000 to 2016. Poor sanitation practices, for example, remain prevalent throughout Malawi, despite progress towards reducing open defecation practices, shown by 53% of all villages being declared open defecation free in 2023 (ODF) [45]. Although policies exist that support implementation of strategies to prevent open defecation practices, the improper use and maintenance of pit latrines remain a concern. Even in regions where pit latrines are widespread, improper construction (faecal sludge containment) or management can lead to considerable risk of contaminating groundwater and surrounding soil with infected faeces. Furthermore, the limited number of hand washing facilities (44%) may lead to food contamination, especially during preparation, handling and processing [46].

In the rural areas of Malawi, pigs are largely kept under free-range systems, reflecting the adoption of low capital-intensive pig farming as a way out of the poverty trap [47]. This results in increased opportunities for exposure to *T. solium* infective material in the environment, especially in areas with poor sanitation [48]. High levels of poverty also increase the risk of *T. solium* infection in the community by restricting access to resources to institute proper pig husbandry and limiting access to healthcare services. While poverty is prevalent in many parts of Malawi, the central and southern regions rank highest according to the multidimensional poverty index at 54.2% [46].

Risk mapping in our study indicated the presence of all three risk factors in central and southern districts of Malawi throughout 2004–2016, while becoming more prevalent in the central districts by 2016. PCC rates in slaughtered animals were highest in one of the central ADDs (Salima), which was bordered by a high-risk district based on the risk factor mapping. The high rate of positive animals is possibly driven by regular meat inspection due to Salima being a major town and tourist site, and due to its size and popularity, Salima acts potentially as a destination for cross-district trade in live pigs, drawing in animals from neighboring high-risk districts. The distribution of NCC patients identified from hospital records clustered around the 2 referral hospitals, except in a few instances. The localization of NCC patients from major urban centers may suggest an association with population density, healthcare access, or other socio-environmental factors, including poor sanitation. The lack of NCC cases identified in likely high-risk areas, based on risk mapping, may be due to the lack of neuro-imaging facilities within the high-risk areas, which represent under-served, resource-limited communities. Furthermore, hospital records did not contain sufficient information to apply the Del Brutto et al. [49] diagnostic criteria to distinguish probable from definitive NCC cases. Only one case at QECH could be classified as definitive, underscoring an additional limitation inherent to retrospective record review, where data are not collected with the diagnostic criteria in mind. Nonetheless, inclusion of hospital records in this study represents an extension to the methodology previously applied in Uganda [17], generating important additional data in a country where there are limited studies available from literature sources.

Combining all data sources into a single map (Fig. 6) provides a more complete picture of the epidemiological situation in Malawi, further highlighting areas where there is a need for further research, including cross-sectional studies of the human and porcine populations. In terms of developing a future national control programme, further research should prioritize the collection of data through population-based surveys in the western and southern parts of the country, which are high risk (brown color on the map), to determine endemic prevalence levels to support the targeting of interventions. Our results also indicate that, despite the existence of public and animal health and sanitation policies and legislation in Malawi, targeted control measures—particularly those focused on water, sanitation, and hygiene (WASH) and combined pig- and human-focused interventions to disrupt the *T. solium* transmission cycle—could be strategically directed toward areas classified as higher risk.

Limitations for the risk factor mapping component include a lack of temporal pig density data, with the study relying on data from one time point based on the Gridded Livestock of the World database in 2007. Incorporating quantitative risk thresholds, based on identifying at which value the proportion of each risk factor reflects a higher risk of facilitating the *T. solium* transmission cycle, would further improve the methodology. Currently, the upper quartile from the MDHS cluster-level values for each risk factor is currently assumed to characterize a high risk, but this represent an arbitrary choice of threshold at present. Additionally, the relative weighting of each risk factor, as all three are currently weighted equally, needs further review to understand whether this assumption requires adjustment.

Despite these limitations, the study on *T. solium* infection in Malawi yielded valuable insights that enhance our understanding of the disease in Malawi. It highlights the urgent need for further research to be conducted. Ultimately, addressing the prevalent risk factors in Malawi will require a comprehensive One Health approach, including improving sanitation and hygiene practices through community education, engagement and climate-resilient WASH infrastructure development. Implementing pig management strategies that limit pig exposure to contaminated areas, in addition to targeted interventions that account for the economic and social challenges faced by impoverished communities, can help bridge the gap in disease control measures.

## Conclusion

This study provides an overview of the epidemiological landscape of *T. solium* infection in Malawi, showing the paucity of population-based studies. Compared to the neighbouring countries like Zambia and Tanzania, there is limited research conducted on *T. solium* in Malawi, hindering a comprehensive understanding of the disease burden and risk factors. Diagnostic disparities, poor sanitation practices, and economic constraints contribute to the persistence of *T. solium* transmission in the region. The congruence of risk factors highlights regions of Malawi where further baseline epidemiological studies can be conducted alongside control measures to reduce the burden of the parasite. We recommend enhanced research efforts prioritizing population-based surveys to obtain baseline prevalence data, pig value chain analysis and embracing One Health approaches to ensure all the stakeholders and sectors are involved in the control efforts.

## Electronic supplementary material

Below is the link to the electronic supplementary material.

Supplementary Material 1

## Data Availability

Data is provided within the manuscript or supplementary information files.
