# Supplementary material for: Spatial and temporal risk mapping of human and porcine Taenia solium infections in Malawi: a systematic review and geostatistical approach
Source: One Health Outlook. 2026 Mar 10;8:19. doi: 10.1186/s42522-026-00199-3 (PMC13003702; doi:10.1186/s42522-026-00199-3)
Supplement: Supplementary file 1 — Supplementary Material 1 [file 42522_2026_199_MOESM1_ESM.docx]

**Supplementary materials**

**Spatial and Temporal Risk Mapping of Human and Porcine *Taenia solium* Infections in Malawi: A Systematic Review and Geostatistical Approach**

Ngwili N^1^, Kachepa U^2^, Salaviriuse A^1,11^, Korir M^1^, Chavula M^3^, Wood C^3^, Chiphwanya J^4^, Kafanikhale H^4,^ Glazer C^5🕆^, Juziwelo L^4🕆^, Pemphero Munkhondia-Phiri^6^, Musaya J^7^, Thomas LF^1,8^*, Dixon MA^9,10^*

^1^Health Program, International Livestock Research Institute, Nairobi, Kenya

^2^Department of Animal Health and Livestock Development, Lilongwe, Malawi

^3^Lilongwe University of Agriculture and Natural Resources, Lilongwe, Malawi

^4^Ministry of Health, Lilongwe, Malawi

^5^University of Liverpool, Liverpool, United Kingdom

^6^Queen Elizabeth Central Hospital, Blantyre, Malawi

^7^Kamuzu University of Health Sciences, Blantyre, Malawi

^8^Royal (Dick) School of Veterinary Studies, University of Edinburgh, Easter Bush Campus, Edinburgh, EH25 9RG, United Kingdom

^9^Department of Infectious Disease Epidemiology and London Centre for Neglected Tropical Disease Research (LCNTDR), Faculty of Medicine, School of Public Health, Imperial College London, London, UK

^10^Unlimit Health, Edinburgh House, London, UK

^11^College of Veterinary Medicine, Animal Resources and Biosecurity, Makerere University, Kampala, Uganda

***^🕆^ Deceased***

****Indicate Joint Senior Author***

**Corresponding authors*:*** [***n.ngwili@cgiar.org***](mailto:n.ngwili@cgiar.org) ***and*** [***m.dixon15@imperial.ac.uk***](mailto:m.dixon15@imperial.ac.uk)

**Additional file Text S1.**

**PICOS (participants, interventions, outcomes, study designs):**

**Participants:** any studies in any type of human or porcine population.

**Interventions:** any interventions where baseline (pre-intervention) infection data are measured.

**Outcomes:** presence (including only the numerator), prevalence and incidence infection markers.

**Study design:** any i) quantitative descriptive or qualitative study; ii) observational study; iii) case report; iv) quasi-experimental/randomised control trial where baseline pre-intervention infection data was collected and reported (see inclusion criteria).

**Inclusion Criteria:**

**Types of Participants:** studies in any type of human or porcine population reporting infection and/or presence (prevalence, incidence) of *T. solium* PCC, HTT and human cysticercosis/NCC.

**Concept:** studies measuring any *T. solium* infection marker (see context and type of studies for more details).

**Context:** relevant studies conducted in Malawi, up to 10 August 2022 (no lower limit).

Type of Studies: any i) quantitative descriptive or qualitative study; ii) observational studies; iii) case reports; iv) quasi-experimental/randomised control trial where baseline pre-intervention infection data was collected and reported. This includes literature/systematic reviews and meta-analyses. Studies using biological samples from Malawi for diagnostic test evaluation will be excluded.

**Language:** no language restrictions are applied.

**Publication Type/Status:** no restrictions were placed on publication type, therefore grey literature consisting of informally published written material including these and medical reports were included in addition to peer-reviewed literature.

**Search Terms**

The search terms were adapted from(Ngowi et al., 2019) and (Ngwili et al., 2023) and edited for the different databases as listed below. The search was conducted on 6^th^ September 2022 at 11am.

**PubMed**

((((((((“Taenia solium” [Mesh]) OR pork tapeworm) OR pork tapeworms) OR tapeworm, pork) OR tapeworms, pork) OR cysticercosis) OR neurocysticercosis)) AND (((Malawi) OR “Republic of Malawi”))

**African Journals Online**

((((((((Taenia solium) OR pork tapeworm) OR (pork tapeworms) OR tapeworm, pork) OR (tapeworms, pork) OR cysticercosis) OR neurocysticercosis)) AND (Malawi)

**Cabdiret**

("Taenia solium" OR "pork tapeworm" OR "pork tapeworms" OR tapeworm OR "pork tapeworms" OR cysticercosis OR neurocysticercosis) AND (Malawi OR "Republic of Malawi")

**OVID Medline**

((((((((“Taenia solium” [Mesh]) OR pork tapeworm) OR pork tapeworms) OR tapeworm, pork) OR tapeworms, pork) OR cysticercosis) OR neurocysticercosis)) AND (((Malawi) OR “Republic of Malawi”))

**Web of Science**

((“cysticercosis”) AND (“Malawi”))

**Cochrane Library – 0 RESULTS**

((“cysticercosis) AND (Malawi”)) OR (“southern Africa”)

**Google Scholar**

We searched for relevant articles obtained from reference lists of articles retrieved through the databases above.

***Additional file Table 1. Prior distributions used for informed prevalence estimation***

| **Study** | **Diagnostic** | **Type of distribution for priors** | **Sensitivity** | **Specificity** |
| --- | --- | --- | --- | --- |
| Banda *et al*. 2025 | Lingual palpation | uniform | 0.161, 0.21 | 0.9, 1.0 |
| Banda *et al*. 2025 | Meat inspection | uniform | 0.221, 0.387 | 0.9, 1.0 |
| Values to specify limits of the unform distributions are based on Braae *et al*. [1] | | | | |

The Bayesian framework for estimating informed prevalence is based on that proposed by Speybroeck *et al*. [2], using the relationship outlined in this paper between “apparent” and “true” prevalence as:

$$p =\pi\times SE+ \left( 1- \pi\right) \times\left( 1-SP \right)$$

Where $p$ is the “apparent” prevalence, $\pi$ is the “true” prevalence, $SE$ is sensitivity and $SP$ is the specificity of the diagnostic. In the Bayesian framework, prior distributions can be specified for the sensitivity and specificity of the diagnostic, however as Speybroeck *et al*. [2] highlight, results will strongly depend on the prior distributions for the diagnostic performance characteristics.

***Additional file Table 2. Study limitations***

| **Reference** | **Title** | **Limitation** |
| --- | --- | --- |
| Bills et al [3] | Cysticercosis producing various neurological presentations in a patient: case report. | Case is not representative of the population at large. Geographic origin of case not described. Individual patient risk factors or environment not explored. |
| Ponnighaus et al [4] | Cutaneous manifestation of cysticercosis | Not much data is given in terms of which specific village patient originated from, nor any data on the timeframe the case study occurred in |
| Kumwenda et al [5] | Differential diagnosis of stroke in a setting of high HIV prevalence in Blantyre, Malawi | Study does not focus on any population and gives a general overview of neurocysticercosis. |
| Uledi SJ [6] | A rare gigantic solitary cysticercosis pseudotumour of the neck | Study is not representative of population and does not state origins of patient |
| Mallewa et al [7] | Overview of the effect and epidemiology of parasitic central nervous system infections in African children. [Review] | Does not provide case study or suspected prevalence of NCC in Malawi |
| Dhesi et al [8] | Imaging in neurocysticercosis. | Case is not representative of the population at large. Geographic origin of case not described. Individual patient risk factors or environment not explored. |
| Heller et al [9] | Case Report: Cysticercosis: Sonographic Diagnosis of a Treatable Cause of Epilepsy and Skin Nodules | Case is not representative of the population at large. Geographic origin of case not described, only point of diagnosis. Individual patient risk factors or environment not explored. |
| Kalata et al [10] | Neurological deterioration in a patient with HIV-associated cryptococcal meningitis initially improving on antifungal treatment: a case report of coincidental racemose neurocysticercosis | Case not representative of general population |
| Stelze et al [11] | Characteristics of people with epilepsy in three Eastern African countries - a pooled analysis. | Investigations focused on evaluation of cryptogenic focal epilepsy |
| Keller et al [12] | Community-level prevalence of epilepsy and of neurocysticercosis among people with epilepsy in the Balaka district of Malawi: A cross-sectional study | only participants that screened positive during the questionnaire underwent further examination, preventing the evaluation of the questionnaires sensitivity and specificity. It was possible some participants were not answering fully due to fear of stigmatization and discrimination resulting in underestimation of seizures |
| Banda L [13] | Prevalence of porcine cysticercosis and risk  Factors. | Low sample size of pig population. |
| Hauser et al [14] | Preliminary assessment of the burden of *Taenia solium* cysticercosis in Malawi | Porcine blood samples for ELISA analysis were stated to have been collected and analysed, however no results were presented in the paper. |

**Additional file Figure 1**


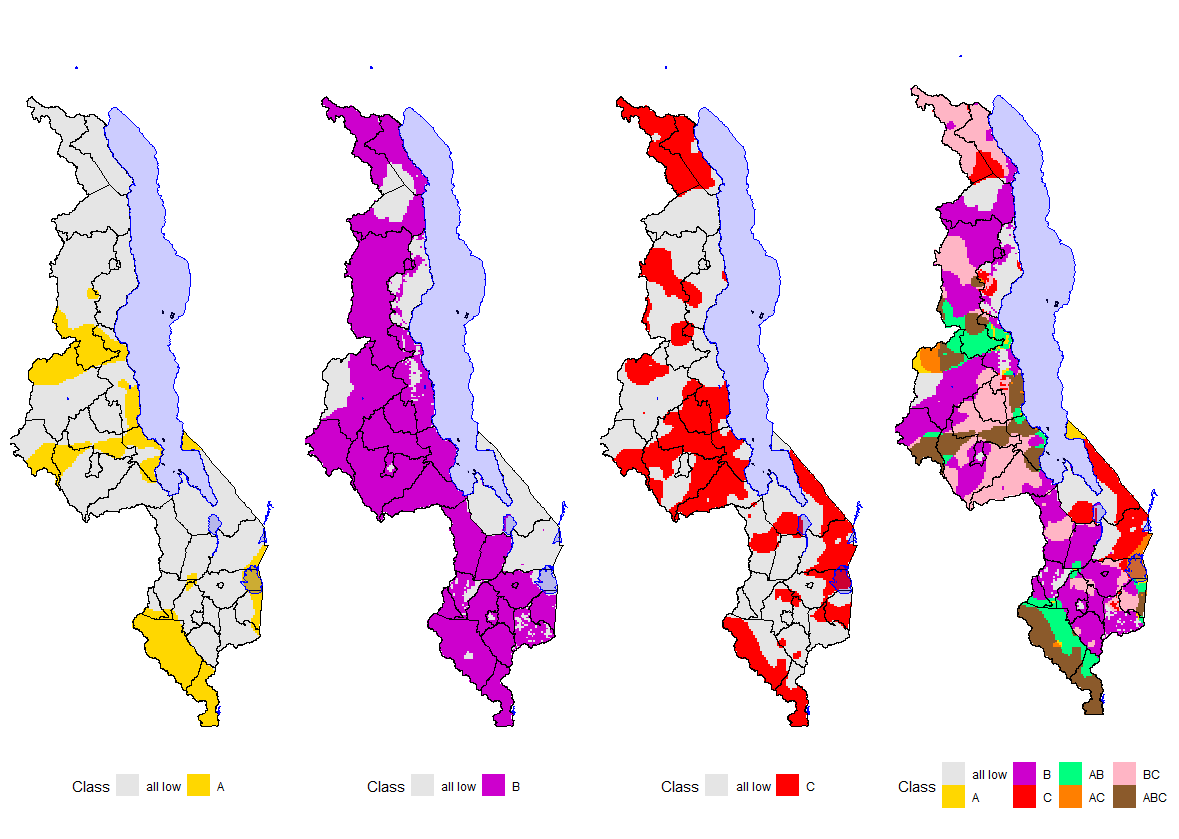


**Supplementary Figure 1. Porcine cysticercosis risk maps for 2000 in Malawi.** Far left panel (high poor sanitation), second left panel (high pig density), second right panel (high poverty) and far right (composite risk). Lake Malawi also plotted.

**Additional file Figure 2**


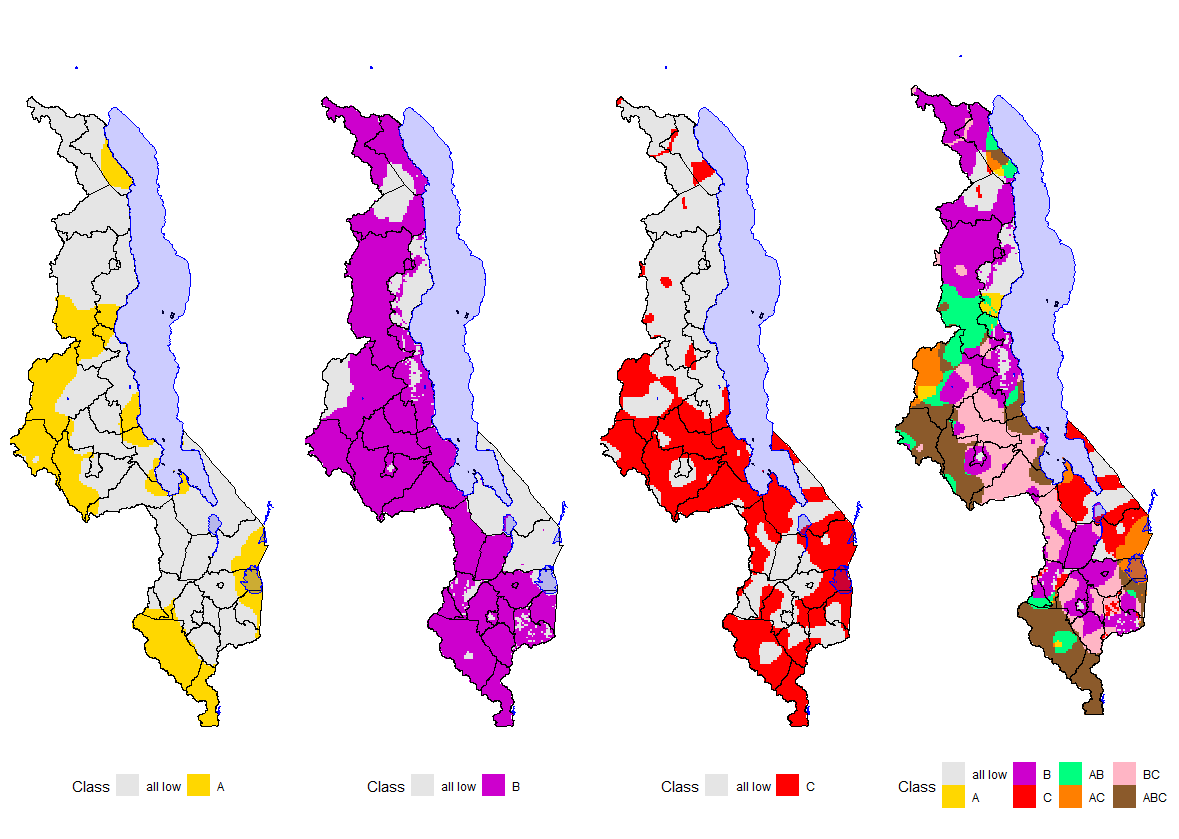


**Supplementary Figure 2. Porcine cysticercosis risk maps for 2004 in Malawi.** Far left panel (high poor sanitation), second left panel (high pig density), second right panel (high poverty) and far right (composite risk). Lake Malawi also plotted.

**Additional file Figure 3**


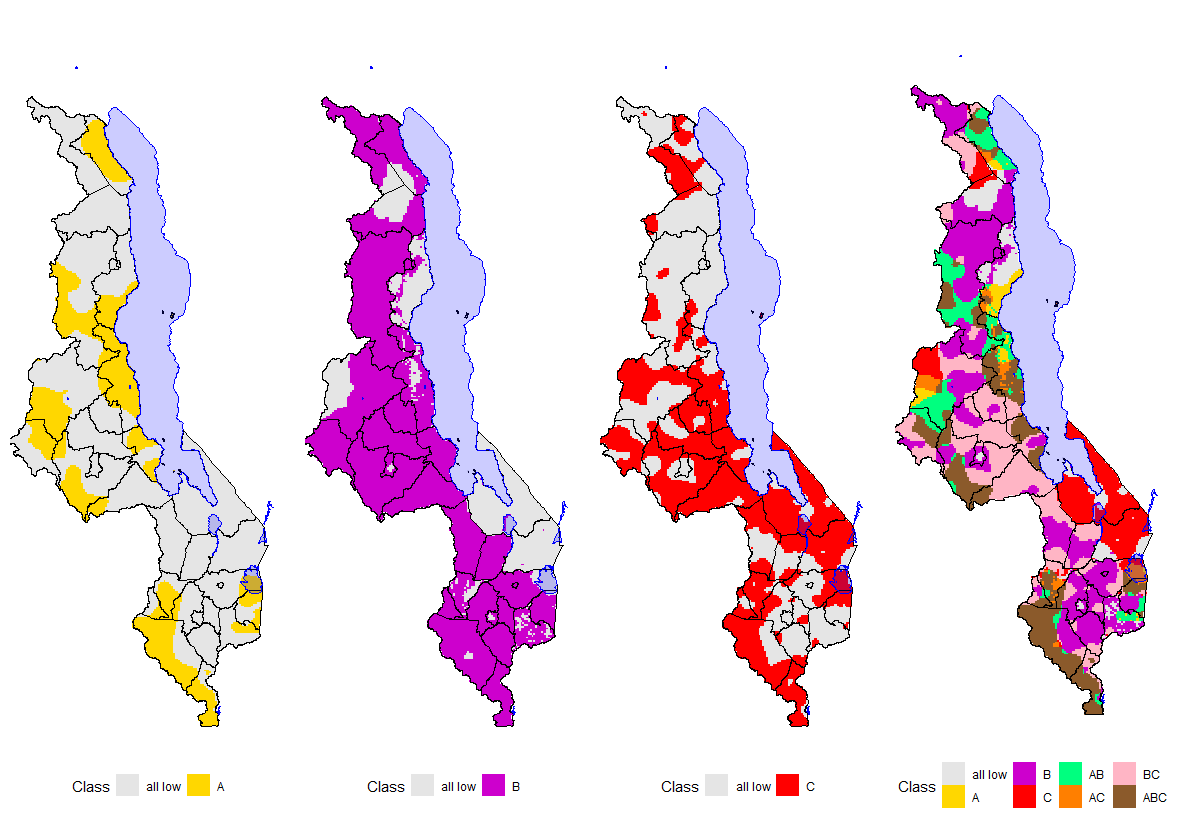
**Supplementary Figure 3. Porcine cysticercosis risk maps for 2010 in Malawi.** Far left panel (high poor sanitation), second left panel (high pig density), second right panel (high poverty) and far right (composite risk). Lake Malawi also plotted.

**Additional file Figure 4**


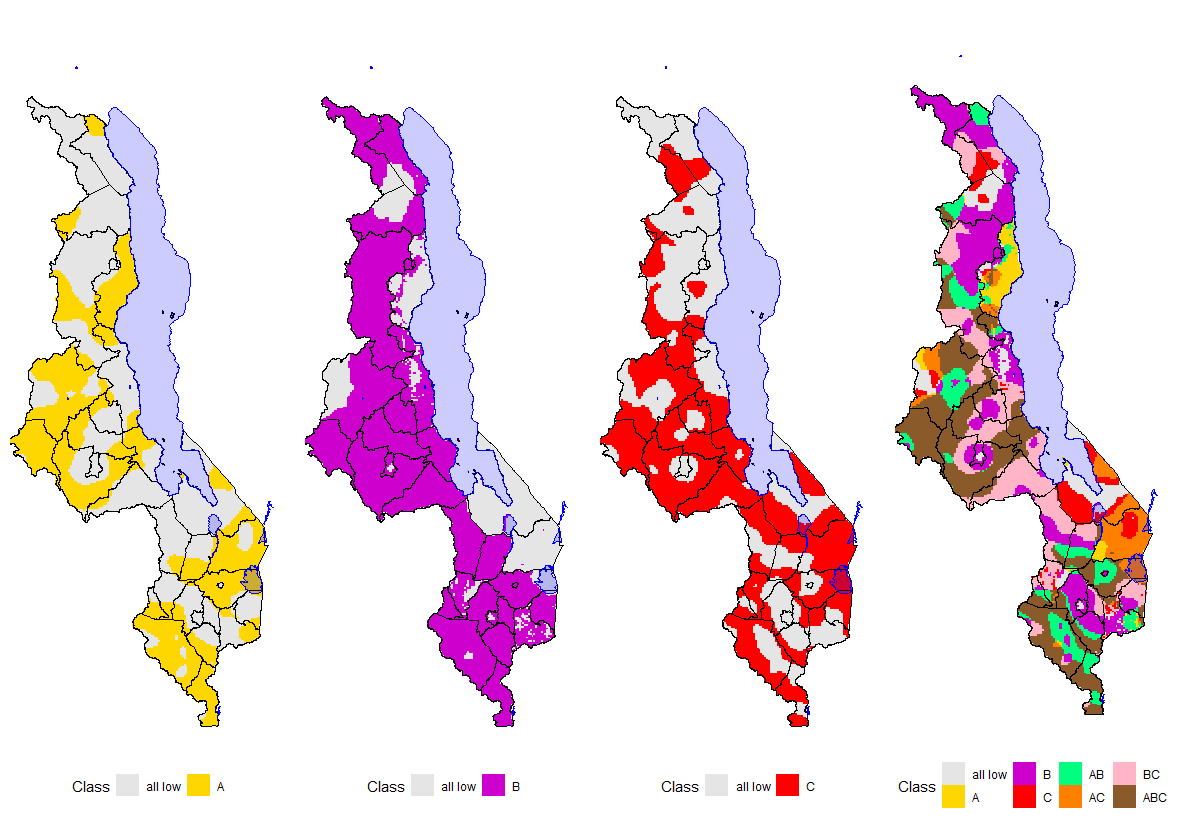


**Supplementary Figure 4. Porcine cysticercosis risk maps for 2016 in Malawi.** Far left panel (high poor sanitation), second left panel (high pig density), second right panel (high poverty) and far right (composite risk). Lake Malawi also plotted.

**Additional file Figure 5**


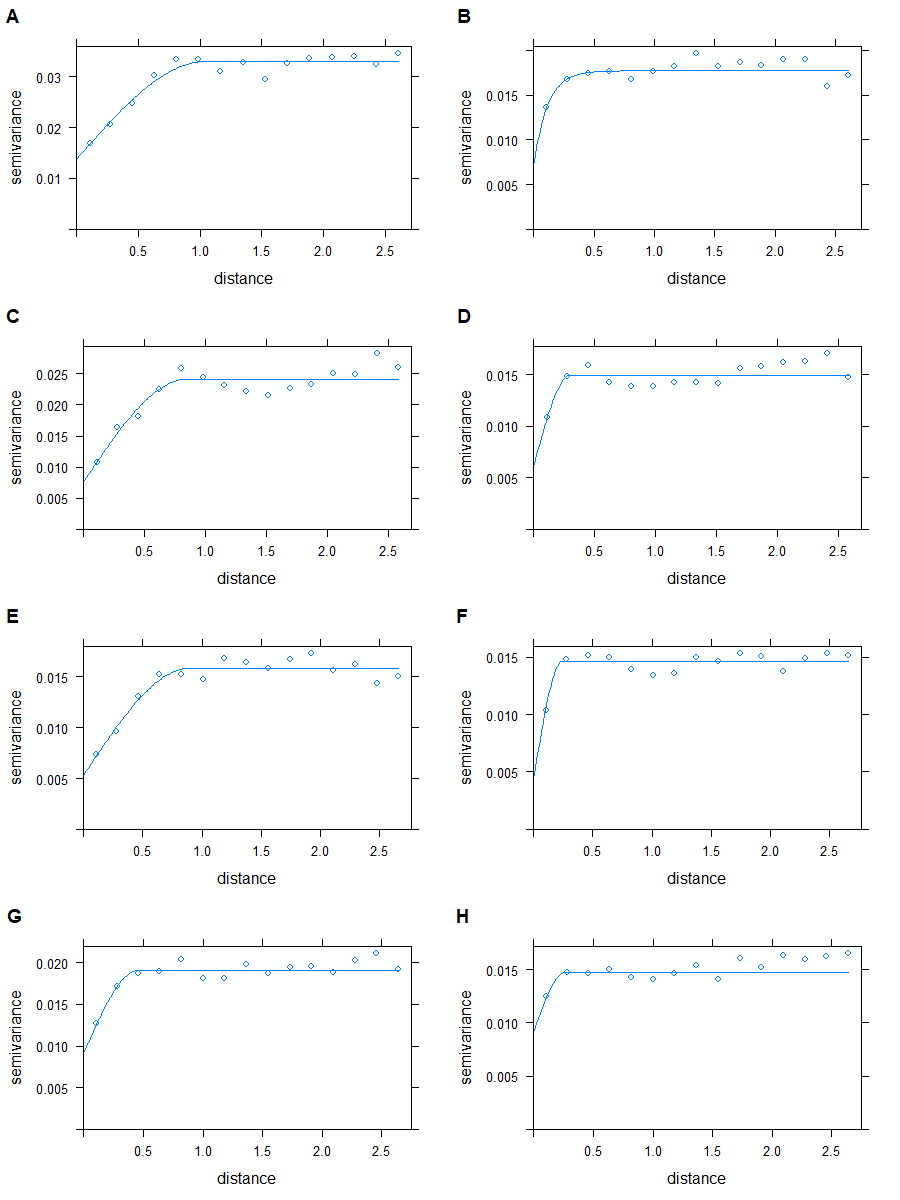


**Supplementary Figure 5. Fitted variograms to DHS cluster level data for each variable and DHS year**. A: sanitation (2000); B: poverty (2000); C: sanitation (2004); D (poverty (2004); E: sanitation (2010); F: poverty (2010); G: sanitation (2016); H: poverty (2016)

**Additional file, Table 2. PRISMA checklist**

| **Section and Topic** | **Item #** | **Checklist item** | **Location where item is reported** |
| --- | --- | --- | --- |
| **TITLE** | | |  |
| Title | 1 | Identify the report as a systematic review. | NA |
| **ABSTRACT** | | |  |
| Abstract | 2 | See the PRISMA 2020 for Abstracts checklist. | Pg. 1-2 |
| **INTRODUCTION** | | |  |
| Rationale | 3 | Describe the rationale for the review in the context of existing knowledge. | Pg. 3 – 4. |
| Objectives | 4 | Provide an explicit statement of the objective(s) or question(s) the review addresses. | Pg. 4. |
| **METHODS** | | |  |
| Eligibility criteria | 5 | Specify the inclusion and exclusion criteria for the review and how studies were grouped for the syntheses. | Appendix (Pg. 2) |
| Information sources | 6 | Specify all databases, registers, websites, organisations, reference lists and other sources searched or consulted to identify studies. Specify the date when each source was last searched or consulted. | Pg. 7, appendix (pp 2– 3) |
| Search strategy | 7 | Present the full search strategies for all databases, registers and websites, including any filters and limits used. | Appendix (pp 2 – 3) |
| Selection process | 8 | Specify the methods used to decide whether a study met the inclusion criteria of the review, including how many reviewers screened each record and each report retrieved, whether they worked independently, and if applicable, details of automation tools used in the process. | Appendix (pp 2 – 3) |
| Data collection process | 9 | Specify the methods used to collect data from reports, including how many reviewers collected data from each report, whether they worked independently, any processes for obtaining or confirming data from study investigators, and if applicable, details of automation tools used in the process. | Appendix (Pg. 6 – 7) |
| Data items | 10a | List and define all outcomes for which data were sought. Specify whether all results that were compatible with each outcome domain in each study were sought (e.g. for all measures, time points, analyses), and if not, the methods used to decide which results to collect. | Appendix (Pg. 3 – 4) |
|  | 10b | List and define all other variables for which data were sought (e.g. participant and intervention characteristics, funding sources). Describe any assumptions made about any missing or unclear information. | Appendix; Supplementary Table 1, pp 3-5 |
| Study risk of bias assessment | 11 | Specify the methods used to assess risk of bias in the included studies, including details of the tool(s) used, how many reviewers assessed each study and whether they worked independently, and if applicable, details of automation tools used in the process. | Appendix (pg.12) |
| Effect measures | 12 | Specify for each outcome the effect measure(s) (e.g. risk ratio, mean difference) used in the synthesis or presentation of results. | Pg. 5. |
| Synthesis methods | 13a | Describe the processes used to decide which studies were eligible for each synthesis (e.g. tabulating the study intervention characteristics and comparing against the planned groups for each synthesis (item #5)). | Tables 2 and 3 |
|  | 13b | Describe any methods required to prepare the data for presentation or synthesis, such as handling of missing summary statistics, or data conversions. | Pg. 6. |
|  | 13c | Describe any methods used to tabulate or visually display results of individual studies and syntheses. | Pg. 5. |
|  | 13d | Describe any methods used to synthesize results and provide a rationale for the choice(s). If meta-analysis was performed, describe the model(s), method(s) to identify the presence and extent of statistical heterogeneity, and software package(s) used. | Table 2 and 3, Figure 2 map |
|  | 13e | Describe any methods used to explore possible causes of heterogeneity among study results (e.g. subgroup analysis, meta-regression). | NA |
|  | 13f | Describe any sensitivity analyses conducted to assess robustness of the synthesized results. | NA |
| Reporting bias assessment | 14 | Describe any methods used to assess risk of bias due to missing results in a synthesis (arising from reporting biases). | NA |
| Certainty assessment | 15 | Describe any methods used to assess certainty (or confidence) in the body of evidence for an outcome. | Pg. 5. |
| **RESULTS** | | |  |
| Study selection | 16a | Describe the results of the search and selection process, from the number of records identified in the search to the number of studies included in the review, ideally using a flow diagram. | Pg. 8. |
|  | 16b | Cite studies that might appear to meet the inclusion criteria, but which were excluded, and explain why they were excluded. | Pg. 8; PRISMA Flow diagram. Figure 1 |
| Study characteristics | 17 | Cite each included study and present its characteristics. | Table 2 and Table 3 (Pg. 9.) |
| Risk of bias in studies | 18 | Present assessments of risk of bias for each included study. | NA |
| Results of individual studies | 19 | For all outcomes, present, for each study: (a) summary statistics for each group (where appropriate) and (b) an effect estimate and its precision (e.g. confidence/credible interval), ideally using structured tables or plots. | Pg. 8 – 9. |
| Results of syntheses | 20a | For each synthesis, briefly summarise the characteristics and risk of bias among contributing studies. | NA |
|  | 20b | Present results of all statistical syntheses conducted. If meta-analysis was done, present for each the summary estimate and its precision (e.g. confidence/credible interval) and measures of statistical heterogeneity. If comparing groups, describe the direction of the effect. | Pg. 8 – 10. |
|  | 20c | Present results of all investigations of possible causes of heterogeneity among study results. | NA |
|  | 20d | Present results of all sensitivity analyses conducted to assess the robustness of the synthesized results. | NA |
| Reporting biases | 21 | Present assessments of risk of bias due to missing results (arising from reporting biases) for each synthesis assessed. | NA |
| Certainty of evidence | 22 | Present assessments of certainty (or confidence) in the body of evidence for each outcome assessed. | Pg. 9 – 10. |
| **DISCUSSION** | | |  |
| Discussion | 23a | Provide a general interpretation of the results in the context of other evidence. | Pg. 18. |
|  | 23b | Discuss any limitations of the evidence included in the review. | Pg. 19. |
|  | 23c | Discuss any limitations of the review processes used. | Pg. 21. |
|  | 23d | Discuss implications of the results for practice, policy, and future research. | Pg. 22. |
| **OTHER INFORMATION** | | |  |
| Registration and protocol | 24a | Provide registration information for the review, including the register name and registration number, or state that the review was not registered. | Pg.4 |
|  | 24b | Indicate where the review protocol can be accessed, or state that a protocol was not prepared. | Appendix (Pg 1-5) |
|  | 24c | Describe and explain any amendments to information provided at registration or in the protocol. | NA |
| Support | 25 | Describe sources of financial or non-financial support for the review, and the role of the funders or sponsors in the review. | Pg. 22. |
| Competing interests | 26 | Declare any competing interests of review authors. | Pg. 23. |
| Availability of data, code and other materials | 27 | Report which of the following are publicly available and where they can be found: template data collection forms; data extracted from included studies; data used for all analyses; analytic code; any other materials used in the review. | Pg. 17. |

*From:*  Page MJ, McKenzie JE, Bossuyt PM, Boutron I, Hoffmann TC, Mulrow CD, et al. The PRISMA 2020 statement: an updated guideline for reporting systematic reviews. BMJ 2021;372:n71. doi: 10.1136/bmj.n71 For more information, visit: <http://www.prisma-statement.org/>

**References**

1. Braae UC, Saarnak CF, Mukaratirwa S, Devleesschauwer B, Magnussen P, Johansen MV. *Taenia solium* taeniosis/cysticercosis and the co-distribution with schistosomiasis in Africa. Parasit Vectors. 2015 Jun 12;8:323. doi: 10.1186/s13071-015-0938-7.
2. Speybroeck, N., Devleesschauwer, B., Joseph, L., & Berkvens, D. (2013). Misclassification errors in prevalence estimation: Bayesian handling with care. *International journal of public health*, *58*(5), 791–795. <https://doi.org/10.1007/s00038-012-0439-9>
3. Bills DC, Symon L. Cysticercosis producing various neurological presentations in a patient: case report. Br J Neurosurg. 1992;6:365–9.
4. Pönnighaus JM, Nkhosa P, Baum HP. Kutane Manifestation der Zystizerkose [Cutaneous manifestation of cysticercosis]. Hautarzt. 2001 Dec;52(12):1098-100. German. doi: 10.1007/s001050170020. PMID: 11910860.
5. Kumwenda J, Mateyu G, Kampondeni S, Van Dam A, Van Lieshout L, Zijlstra E. Differential diagnosis of stroke in a setting of high HIV prevalence in Blantyre, Malawi. Malawi Med J. 2006;17.
6. Uledi SJ. A rare gigantic solitary cysticercosis pseudotumour of the neck. J Surg Case Rep. 2010 Nov 1;2010(9):5. doi: 10.1093/jscr/2010.9.5.
7. Mallewa M, Wilmshurst JM. Overview of the effect and epidemiology of parasitic central nervous system infections in African children. Semin Pediatr Neurol. 2014 Mar;21(1):19-25. doi: 10.1016/j.spen.2014.02.003.
8. Dhesi B, Karia SJ, Adab N, Nair S. Imaging in neurocysticercosis. Pract Neurol. 2015;15:135–7.
9. Heller T, Wallrauch C, Kaminstein D, Phiri S. Case Report: Cysticercosis: Sonographic Diagnosis of a Treatable Cause of Epilepsy and Skin Nodules. Am J Trop Med Hyg. 2017 Dec;97(6):1827-1829. doi: 10.4269/ajtmh.17-0257.
10. Kalata N, Ellis J, Benjamin L, Kampondeni S, Chiodini P, Harrison T, et al. Neurological deterioration in a patient with HIV-associated cryptococcal meningitis initially improving on antifungal treatment: a case report of coincidental racemose neurocysticercosis. BMC Infect Dis. 2021;21.
11. Stelzle D, Schmidt V, Keller L, Ngowi BJ, Matuja W, Escheu G, Hauke P, Richter V, Ovuga E, Pfausler B, Schmutzhard E, Amos A, Harrison W, Kaducu J, Winkler AS. Characteristics of people with epilepsy and Neurocysticercosis in three eastern African countries-A pooled analysis. PLoS Negl Trop Dis. 2022 Nov 7;16(11):e0010870. doi: 10.1371/journal.pntd.0010870. Erratum in: PLoS Negl Trop Dis. 2023 Jan 26;17(1):e0011101. doi: 10.1371/journal.pntd.0011101.
12. Keller L, Stelzle D, Schmidt V, Carabin H, Reinhold AK, Keller C, et al. Community-level prevalence of epilepsy and of neurocysticercosis among people with epilepsy in the Balaka district of Malawi: A cross-sectional study. PLoS Negl Trop Dis. 2022;16.
13. Banda L, Bailey J, Kristina R, Mutua F, Melaku T. Prevalence and associated risk factors of porcine cystcercosis. 2019.
14. Hauser M, Amos R, Cargill T, Chikungwa P, Njunga G, Chinombo D, et al. Preliminary assessment of the burden of *Taenia solium* cysticercosis in Malawi. University of Copenhagen.
15. Page MJ, McKenzie JE, Bossuyt PM, Boutron I, Hoffmann TC, Mulrow CD, et al. The PRISMA 2020 statement: an updated guideline for reporting systematic reviews. BMJ 2021;372:n71. doi: 10.1136/bmj.n71 For more information, visit: http://www.prisma-statement.org/
